# Supplementary material for: Polymer-Dispersed Cholesteric Liquid Crystal under Homeotropic Anchoring: Electrically Induced Structures with λ1/2-Disclination
Source: Polymers (Basel). 2022 Apr 2;14(7):1454. doi: 10.3390/polym14071454 (PMC9002932; doi:10.3390/polym14071454)
Supplement: Supplementary file 1 [file polymers-14-01454-s001.zip › Supplimentary.pdf]

Supplementary Information

Electrically induced structures with $\lambda^{1/2}$-disclination in cholesteric droplets underthe homeotropic boundary conditions

Anna P. Gardymova[1,2*], Mikhail N. Krakhalev[1,2], VladimirYu. Rudyak[3], Vadim A. Barbashov[4] and Victor Ya. Zyryanov[1]

[1]*Kirensky Institute of Physics, Federal Research Center KSC SBRAS, 50/38 Akademgorodok, Krasnoyarsk, 660036, Russia.*

[2]*Institute of Engineering Physics and Radio Electronics, SiberianFederal University, 79 Svobodny Pr., Krasnoyarsk, 660041, Russia.*

[3]*Faculty of Physics, Moscow State University, 1/2 LeninskiyeGory, Moscow, 119991, Russia.*

[4]*Lebedev Physical Institute of the Russian Academy of Sciences,53 Leninskiy prospekt, Moscow, 119991, Russia.*

Corresponding author: A.P. Gardymova, e-mail: gard@iph.krasn.ru

Supplementary Figure S1, Figure S2, Movie

Figure S1| CLC droplets before and after the action of an electric field.

Figure S2| CLC droplets with structure with $\lambda^{+1/2}$-disclination or $\lambda^{-1/2}$-disclination.

Movie| Transformation of the CLC droplet structure with a stepwise decrease in applied voltage.

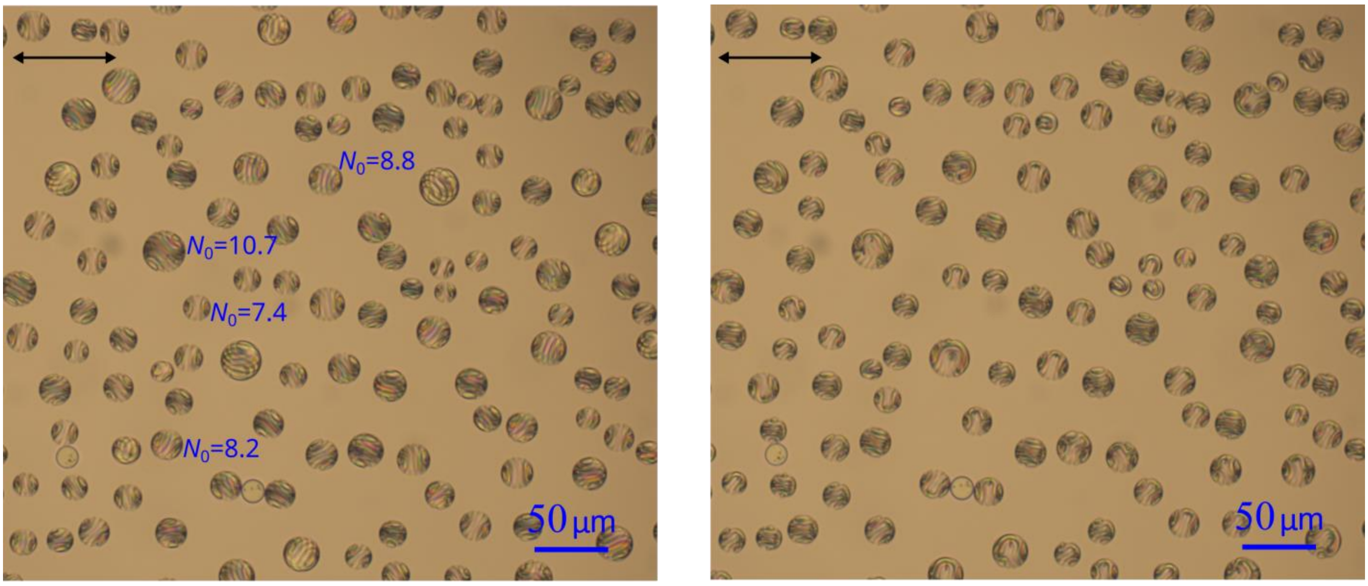

a b

Figure S1| CLC droplets with structure with the bipolar distribution of the helix axis observed before the action of an electric field (a) and with structure with $\lambda^{+1/2}$-disclination forming after the action of an electric field (b).

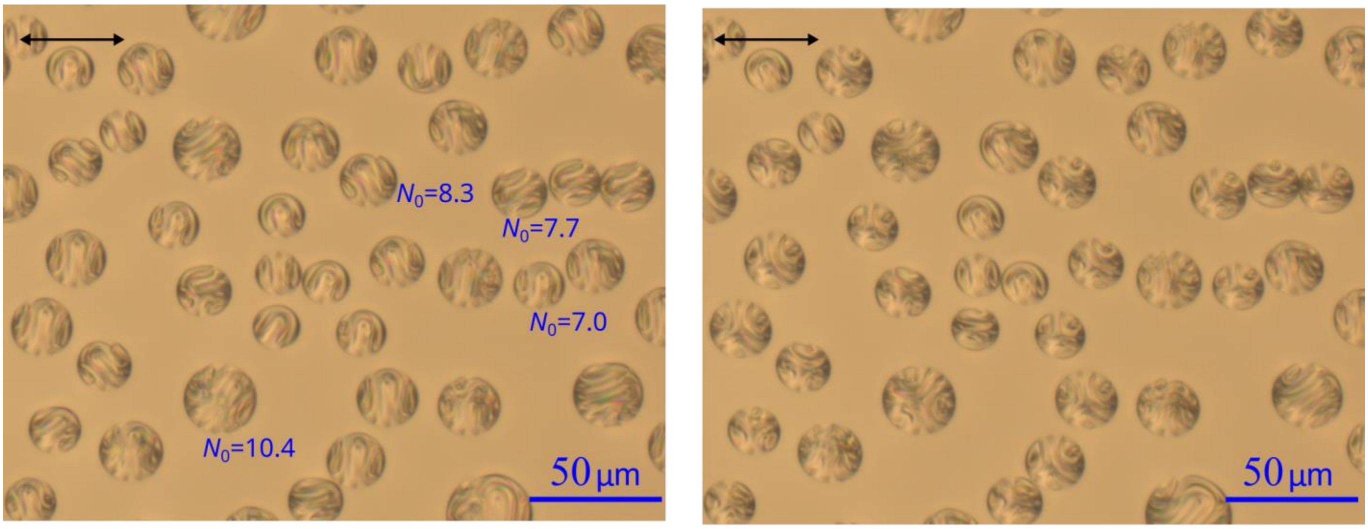

a b

Figure S2| CLC droplets with the structures with $\lambda^{+1/2}$-disclination (a) and with $\lambda^{-1/2}$-disclination formed after the action of an electric field.
